# Draft Genome Sequence of *Bacillus cereus* LCT-BC25, Isolated from Space Flight

**DOI:** 10.1128/genomeA.00667-13

**Published:** 2014-01-02

**Authors:** Xuelin Zhang, Tong Wang, Longxiang Su, Lisha Zhou, Tianzhi Li, Junfeng Wang, Yan Liu, Xuege Jiang, Chunyan Wu, Changting Liu

**Affiliations:** Nanlou Respiratory Diseases Department, Chinese PLA General Hospital, Beijing, Chinaa; BGI-Shenzhen, Shenzhen, Chinab; Medical College, Nankai University, Tianjin, Chinac

## Abstract

*Bacillus cereus* strain LCT-BC25, which was carried by the Shenzhou VIII spacecraft, traveled in space for about 398 h. To investigate the response of *B. cereus* to space environments, we determined the genome sequence of *B. cereus* strain LCT-BC25, which was isolated after space flight.

## GENOME ANNOUNCEMENT

To investigate the response of *Bacillus cereus* to space environments, this bacterium was carried into space for 398 hours by the Shenzhou VIII spacecraft. After the Shenzhou VIII spacecraft landed, *B. cereus* strain LCT-BC25 was selected as a clone strain; it showed a lower growth rate than and changes in metabolism compared to the ground-control strain, LCT-BC244. In addition, strain LCT-BC25 produced a significantly smaller inhibition zone (1.3 cm) than LCT-BC244 (2.6 cm) in the LB plates supplemented with amikacin (1).

Whole-genome sequencing was performed using the Illumina HiSeq 2000 (Illumina, Inc.) by generating paired-end libraries (350 bp and 6 kb). The read length was 90 bp for both libraries, from which 580 Mb and 333 Mb of high-quality data were generated, respectively. The paired-end reads were first de novo assembled using SOAPdenovo version 1.05, and then the scaffolds were connected according to 6-kb paired-end relationships. The coding sequences (CDS) were predicted by using Glimmer version 3.02. Homologous comparison of all of the genes was performed by BLAST with the NCBI nonredundant public database, KEGG, COG, Swiss-Prot, TrEMBL, and GO for function annotation. The rRNAs and tRNAs were identified using RNAmmer and tRNAscan-SE 1.21, respectively. Other noncoding RNAs, including microRNA (miRNA), small RNA (sRNA), and small nuclear RNA (snRNA), were analyzed by using the Infernal software and the Rfam database. Meanwhile, transposons were determined based on the Repbase transposable elements library or by using RepeatMasker and Repeat-ProteinMasker. Tandem repeats were predicted by use of TRF software.

We obtained 7 scaffolds consisting of 43 contigs with a total length of 5,154,512 bp, and the G+C content was determined to be 35.31%. From the genome composition analysis results, we found that the genome contained 5,257 genes with an average length of 837 bp, and the total length of genes was 4,401,039 bp, which makes up 85.38% of the genome. Among the whole-gene sets, 2,765 CDSs were involved in the 22 functional COG groups and 2,908 CDSs were involved in the 34 metabolic pathway KEGG groups. We found 37 tRNAs with a total length of 2,866 bp, which make up 0.0556% of the genome. In addition, 2 rRNAs and 4 sRNAs were also determined. Furthermore, there were 171 transposons and 324 tandem repeats found in the genome.

### Nucleotide sequence accession number.

This whole-genome shotgun project of *Bacillus cereus* LCT-BC25 has been deposited at DDBJ/EMBL/GenBank under the accession ATHM00000000. The version described in this paper is the first version.
